# Investigation of Differences in Allostatic Load among Black Men by Level of Educational Attainment: High School Graduates Experience the Highest Levels of Stress

**DOI:** 10.3390/ijerph19063580

**Published:** 2022-03-17

**Authors:** Charles R. Rogers, Justin X. Moore, Danielle R. Gilmore, Ethan Petersen, Ellen Brooks, Carson Kennedy, Roland J. Thorpe

**Affiliations:** 1 Department of Family & Preventive Medicine, School of Medicine, University of Utah, Salt Lake City, UT 84108, USA; ethan.petersen@hsc.utah.edu (E.P.); ellen.brooks@utah.edu (E.B.); u1068835@utah.edu (C.K.); 2 Cancer Prevention, Control & Population Health, Georgia Cancer Center, Department of Medicine, Medical College of Georgia, Augusta University, Augusta, GA 30912, USA; jusmoore@augusta.edu; 3 Trachtenberg School of Public Policy and Administration, George Washington University, Washington, DC 20052, USA; danielleg@email.gwu.edu; 4 Program for Research on Men’s Health, Hopkins Center for Health Disparities Solutions, Johns Hopkins University, Baltimore, MD 21205, USA; rthorpe@jhu.edu

**Keywords:** allostasis, health equity, men’s health, socioeconomic factors, social determinants of health

## Abstract

Allostatic load (AL)—the biological assessment of long-term exposure to stress—may explain mortality-rate disparities among non-Hispanic Black (Black) men. We aimed to investigate AL among Black men with equivalent education status after controlling for income. A cross-sectional study was employed to investigate AL among 4113 Black men who participated in the National Health and Nutrition Examination Survey between 1999–2018. A summation of 8 biomarker factors were used to compute AL, differences in socio-demographic characteristics by education status were evaluated, and health behaviors that may influence AL were examined. To determine the high-risk thresholds for each AL component, we examined each component’s distribution among NHB men for whom complete biomarker data were available in the NHANES sample. High-risk thresholds were determined as either (1) above the 75th percentile for body mass index (BMI), diastolic blood pressure (DBP), glycated hemoglobin, systolic blood pressure (SBP), total cholesterol, and serum triglycerides; or (2) below the 25th percentile for serum albumin and serum creatinine. Modified Poisson regression models were used to estimate prevalence ratios and their associated 95% confidence intervals for high AL risk while adjusting for potential confounders. Black men with a high school diploma/GED had a greater prevalence of high AL compared with Black men who had other levels of education, and a slightly higher prevalence of high AL compared with Black men who had less than a high school education. Black men with college degrees had a lower prevalence of high AL than Black men with the lowest levels of educational attainment. Researchers must further examine the hidden costs stemming from the interplay between discrimination associated with being Black in America and systemic racism in the educational system—which may be preventing Black men from achieving optimal health.

## 1. Introduction

Non-Hispanic Black (Black) men experience disproportionately higher rates of poor health and chronic illness (e.g., diabetes, hypertension, stroke) than non-Hispanic White (White) men [1,2]. Allostatic load (AL), a measure of the physiological effect of chronic stress [2,3,4], may help to explain mortality and morbidity disparities between Black and White men [1]. Chronic stress results from pervasive discrimination and social disadvantage, experiences more prevalent in everyday life for Black men than White men [2,5]. AL highlights the effects of cumulative exposure to chronic stress–induced physiologic changes, which can lead to accelerated aging, an idea formulated in the weathering hypothesis [2,3,4]. Additionally, Black men are more likely to live in environment with higher levels of lead, which further contributes to AL as exposure is linked to chronic stress and a host of chronic conditions including cardiovascular disease mortality [6,7].

Conditions contributing to chronic stress are attributable to social determinants of health (SDoH), factors such as income, education, childhood experiences, and racism that influence quality of life, health outcomes, and risk [8,9]. Substantial evidence shows that Black men experience poor health outcomes due to SDoH [10,11]. AL can be a useful measure of the impact of SDoH on the health of Black men [3,11], who experience higher AL than White men [2,12,13,14]. Although the relationship between AL, structural racism, and SDoH is complex [15,16], clear evidence exists that higher AL leads to poor health outcomes and higher all-cause mortality [17,18].

Socioeconomic status (SES) is a SDoH that contributes significantly to an individual’s biological risk profile and AL [14]. Hickson et al. [19] suggest that, compared with other SDoH, SES more robustly influences AL in Black individuals. Multiple studies have demonstrated, however, that Black men have higher AL than White men even after controlling for SES [1]. Among those with high SES, Black individuals do not experience reduced AL commensurate with that seen in White individuals, indicating that stressors such as systemic, structural, and interpersonal racism may profoundly affect Black individuals’ health [1,3,20,21,22].

### Study Purpose and Hypothesis

Education level, a SDoH, has been found to be inversely related to AL [23,24]. However, while increases in education level correspond to reduced AL, the alleviating effect of education may be diminished in Black compared with White individuals [24]. Moreover, studies involving mono- and dizygotic twins found that education alone did not protect against high AL [25], suggesting that further investigation of the relationship between education, other SDoH, and AL is warranted.

Differences in Black men’s economic and social conditions may partly explain their higher AL and comparably worse health status [1,3,12,13]. In a race-conscious society, a higher education level and household income may interact to create higher AL among Black men who work in predominantly White geographic areas [26]. Socio-demographic changes observed with increasing social mobility may contribute to perceived racial discrimination, lack of social support, and feelings of isolation [27,28]. Additionally, while life expectancy in the US has continued to increase, Black men still lag significantly behind both their White peers and Black female counterparts. Despite advances in health care and increased educational opportunities the Black-White life expectancy gap has only continued to widen, especially among males [29,30].

Although an extensive literature explores the influence of multiple SDoH on AL in Black men, a need remains to thoroughly examine the relationship between AL and individual SDoH, such as education level. Accordingly, the purpose of this study was to investigate whether AL is associated with education status in Black men, after controlling for income. We hypothesized that Black men with advanced education would have higher AL even after controlling for the potential material benefits accompanying advanced education and higher income.

## 2. Materials and Methods

### 2.1. Data Source and Study Population

We performed a cross-sectional study among Black men who participated in the National Health and Nutrition Examination Survey (NHANES), a survey research program conducted by the National Center for Health Statistics (NCHS) to assess and track changes over time in the health and nutritional status of adults and children in the United States. To generate accurate statistics, NHANES intentionally oversamples persons aged 60 years and older and Hispanic and Black individuals representative of the U.S. population. Oversampling traditionally underrepresented populations facilitates weighted analyses to generate more-reliable generalizable estimates [31,32]. The NHANES weighted sample is considered representative of the US civilian noninstitutionalized population [33]. NHANES includes demographic, socioeconomic, and health-related questionnaires as well as clinical measures of blood pressure, fasting blood glucose, triglycerides and HDL cholesterol, and self-reported medication use. We used NHANES data for the years 1999 through 2018, with congruent data on the component variables of AL [34], education, and household income. In consideration of the analytic guideline changes that went into effect in 2011, we appropriately modified statistical weights and accounted for primary sampling units and strata within analyses [32,33].

Following the NCHS guidelines for NHANES data [32], we analyzed data from the fasting subsample. We included all Black men aged 18 and older with available data on AL components (i.e., those in the NHANES fasting subsample for biomarkers) and with no missing information on educational attainment and poverty-to-income (PIR) ratio, a total of 4113 Black men among 41,169 NHANES participants. No values for other covariates were missing. We did not include Black women in this study because we wished to focus on the health and well-being of Black men, an understudied population in AL research. Additionally, Black men have unique lived experiences due to their demonization and criminalization [35], perpetuating discrimination and warranting separate and intragroup analysis.

### 2.2. Ethical Approval Statement

This study was exempt from approval by the Institutional Review Boards of the University of Utah, the Medical College of Georgia at Augusta University, and Johns Hopkins University because the datasets used are publicly available.

### 2.3. Dependent Variable: Allostatic Load

Most definitions of AL incorporate biomarker measures from three categories of physiologic functioning: cardiovascular, metabolic, and immune system. Absent a consensus definition, we defined AL using the Geronimus et al. [3] and Mays et al. [36] taxonomies. Allostatic load components included body mass index (BMI), diastolic blood pressure (DBP), glycohemoglobin (hemoglobin A1c), systolic blood pressure (SBP), total cholesterol, serum triglycerides serum albumin, and serum creatinine. To determine the high-risk thresholds for each AL component, we examined each component’s distribution among Black men for whom complete biomarker data were available in the NHANES sample. High-risk thresholds were determined as either (1) above the 75th percentile for BMI, DBP, glycohemoglobin, SBP, total cholesterol, and serum triglycerides; or (2) below the 25th percentile for serum albumin and serum creatinine. Each Black received a score of either 1 (high risk) or 0 (low risk) for each component variable based on the cut-points shown in Table A1. We summed individual components to calculate a total AL score ranging from 0 to 8 and categorized participants with an AL score of 4 or higher as having high AL [36,37].

### 2.4. Independent Variable and Covariates

Our primary independent variable of interest was educational attainment, which we categorized into four groups: (1) less than high school completion; (2) high school completion or equivalent; (3) some college or an associate’s degree; and (4) college graduate or above. PIR was calculated as the ratio of total household income to poverty threshold values. Persons reporting no income were assigned a zero value for PIR. PIR values of less than 1 are considered below the official poverty line; greater than 1, above the poverty level; and near 5, very high income [38].

We evaluated health characteristics that may influence AL score, including smoking status, and health conditions and comorbidities. Smoking status was categorized as a discrete, mutually exclusive three-level variable: never smokers (fewer than 100 lifetime cigarettes smoked); past smokers (at least 100 lifetime cigarettes smoked, not current smokers); and current smokers (at least 100 lifetime cigarettes smoked, still smoking). We included responses to the survey question “Has a doctor or other health professional ever told you that you had … (cancer, angina, congestive heart failure [CHF], or heart attack)?” as comorbidities and examined them as discrete binary variables (i.e., yes or no for each condition).

### 2.5. Statistical Analysis

Categorical variables were presented as weighted row percentages along with mean and associated standard errors. We compared characteristics (i.e., descriptive statistics) by educational attainment levels using Rao-Scott Chi-Square tests for categorical variables and weighted Wald F-tests for continuous variables. The primary outcome of interest was the prevalence of high AL, presented as prevalence ratios (PRs). We performed several modified Poisson regression models to estimate high AL risk, adjusting for potential confounders, including age, PIR, smoking status, and any history of cancer, congestive heart failure, or heart attack. We additionally performed weighted generalized linear models with a negative binomial distribution associating the mean AL score with educational levels adjusting for the aforementioned confounders. Estimates derived from modified Poisson regression are presented as PRs and associated 95% confidence intervals (CIs), and estimates derived from negative binomial regression models are presented as mean estimates and associated 95% CIs. We assessed the collinearity between family PIR and education by calculating the correlation and variance inflation factor (VIF). There was no evidence of collinearity between family PIR and education as VIF was 1.0 and the correlation was 0.397. We performed secondary analyses to examine a multiplicative interaction between education and family PIR on mean AL. To account for the complex sampling design, analyses were performed using NHANES-generated sampling statistical weights. We estimated the variance for point estimates within Poisson regression utilizing the delete-1 jackknife (resampling) method [39,40,41,42,43,44]. We used SAS software version 9.4 (copyright © 2013 SAS Institute Inc., Cary, NC, USA) for all analyses.

## 3. Results

### 3.1. Study Population Characteristics

Of the 4113 Black men included in this analysis (Table 1), the largest single group reported attaining some college or an Associate’s degree (30.1%), followed by high school completion or equivalent (29.5%), less than high school completion (25.4%), and college graduate or above (15.0%). The mean age of all Black men was 42.4 years (SE = 0.3); more than 1 out of 4 men (27.1%) were aged 18 to 29 years. Black male college graduates were older on average (mean age 44.8 years, SE = 0.5, *p* value < 0.01) and had higher mean PIR (3.62, SE = 0.03, *p* value < 0.01) and a lower prevalence of current smoking status (12.6%) and ever heart attack (2.7%) when compared to Black men with other education levels. Among Black men, mean allostatic load scores were generally higher for men with less than high school completion (2.15, SE = 0.05) compared to other educational attainment levels (2.01, 1.98, 2.05, respectively), though non-significant (*p* value = 0.14).

### 3.2. Prevalence of High Allostatic Load

Black men with high school completion or equivalent had a higher prevalence of high AL (19.2%) than Black men with other education levels. In the unadjusted model, Black men that were college graduates had a 3% lower prevalence of high AL compared with Black men with less than high school completion (Table 2: crude PR = 0.97, 95% CI = 0.93–1.01). Black men that had some college or Associate’s degree had a 2% lower prevalence of high AL compared with Black men with less than high completion (crude PR = 0.98, 95% CI = 0.97–0.99). However, Black men with high school completion or equivalent had a slightly higher prevalence of high AL than those with less than high school completion (crude PR = 1.01, 95% CI = 1.00–1.03).

In age-adjusted-only models, Black male college graduates (Model 1 PR = 0.87, 95% CI = 0.84–0.90) had a 13% lower prevalence of high AL than Black men with less than high school completion. However, Black men with high school completion (Model 1 PR = 1.09, 95% CI = 1.07–1.11) or some college or Associate’s degree (Model 1 PR = 1.03, 95% CI = 1.01–1.04) had a higher prevalence of high AL compared to Black men with less than high school completion.

In the further PIR-adjusted model, we identified a significant relationship between high AL and high AL. Black men that were college graduates (Model 2 PR = 0.92, 95% CI = 0.89–0.96) had an 8% lower prevalence of high AL compared to Black men with less than high school completion. However, Black men with high school completion or equivalent (Model 2 PR = 1.10, 95% CI = 1.08–1.12) had a 10% higher prevalence of high AL compared to Black men with less than high school completion. Black men with some college or an Associate’s degree (Model 2 PR = 1.06, 95% CI = 1.04–1.08) had a 6% higher prevalence of high AL than compared to Black men with less than high school completion. In sequential and fully adjusted models (additionally adjusted for smoking status and comorbidities), we observed that Black male college graduates had a 10% lower prevalence of high AL than Black men with less than high school completion (Model 3 PR = 0.90, 95% CI = 0.87–0.93).

### 3.3. Mean Estimates for Allostatic Load Scores

We additionally examined the relationship between educational levels and mean scores for allostatic load among Black men. We observed that Black male college graduates (Table 3: mean AL = 2.16, 95% CI = 2.04–2.28) and Black men without a high school education (mean AL = 2.16, 95% CI = 2.08–2.25) had similar unadjusted mean allostatic load scores. However, after adjustments for age, income, smoking status, and other comorbidities with observed a U-shaped relationship between educational attainment and mean allostatic load scores; i.e., less than high school (mean AL = 2.19, 95% CI = 2.10–2.29), high school/GED (mean AL = 2.03, 95% CI = 1.94–2.12), some college or associate’s degree (mean AL = 1.97, 95% CI = 1.89–2.07), and college degree (mean AL = 2.06, 95% CI = 1.94–2.18).

## 4. Discussion

We investigated whether AL is associated with education status in Black men, after controlling for income. Our results indicate that advanced educational attainment (i.e., college degree or more) has a protective effect against high AL for Black men, but this effect is not consistent across incremental increases in education level. We observed that Black male college graduates had lower prevalence of high allostatic load when compared to Black men with less than high school completion. However, Black men with intermediate education (i.e., high school/GED and some college or Associates degree) level were more likely to have high allostatic load when compared to Black men with less than high school education. This remained true after controlling for factors such as age, PIR, and chronic health conditions associated with elevated AL.

While higher education levels are understood to generally confer a higher SES, Black men have been shown to experience higher AL compared with White men even after controlling for education [1,45]. Although lower SES is a risk factor for higher AL [14], studies have shown that SES differences alone cannot explain higher AL [1,3]. These findings suggest that the social construct of race leads to differential benefits from education and SES for Black men compared with White men.

Men with higher education levels are more likely to have health insurance, better self-reported health, and fewer chronic conditions, and to live longer than their less-educated peers [46,47]. Our results showed that Black male college graduates tended to have lower AL than those with less than high school completion. This suggests that the effects of other documented factors shown to significantly influence AL regardless of education level (e.g., poverty, systemic and interpersonal racism, access to preventive healthcare and healthy foods, chronic stress) may be weaker in this highly educated sample [3,11,14,24,48,49,50,51,52]. One plausible reason for this observation is that Black men without a college education may endure additional stress because their educational attainment may result in social marginalization and lack of economic opportunity, which may in turn contribute to poor health outcomes and disproportionate mortality rates [35]. This difference in exposure to factors that increase AL among Black men requires further investigation.

We hypothesized that, even after controlling for the material benefits accompanying advanced education and higher income, Black men with advanced education would have a higher AL. After adjusting for confounders, however, we found that Black male college graduates had a lower prevalence of high AL than Black men with the lowest education levels (Table 2). By contrast, we found that Black men with intermediate education levels (i.e., high school completion or equivalent or some college or an associate’s degree) had elevated PR for high AL compared with both college graduates and men with less than high school completion (Table 2). Black men with intermediate education levels also showed slightly elevated PR for an AL greater than 4 after adjusting for age, PIR, smoking history, CHF, and heart attack. This distribution of elevated AL is a unique finding that warrants investigation of other factors that may contribute to high AL among Black men, particularly those with intermediate education levels.

To our knowledge, this is the first study to investigate whether AL is associated with education status among Black men, after controlling for income. This work underscores the impact of education on the health of Black men and suggests that other factors may drive high AL in this population.

While our findings highlight the socioeconomic benefits of education [46,47] and confirm the health-education gradient [46], they also suggest that the expected protective effect of education does not extend to all Black men—specifically, that intermediate education levels do not protect Black men against higher AL compared with men with less than high school completion. While higher education levels often correspond with lower AL [23]—a trend demonstrated consistently among White individuals [23,24], our results add to the literature suggesting that education alone is not consistently protective for Black men [25]. Higher AL scores among Black men of lower education status are consistent with the findings of other studies that have outlined the inverse relationship between education and AL [23,24].

Noteworthy effects have also been found across other health outcomes among Black men. For example, higher household income has been associated with a higher risk for major depressive disorder [27] and a higher education level with more-significant suicidal ideation [53]. The latter finding underscores the need for culturally sensitive suicide-prevention programs and additional interventional research that accounts for education and income gradients in studies of depressive symptoms among Black men.

One plausible explanation for the apparent absence of education’s protective effect against AL in Black men involves changing neighborhood demographics and sociocultural context as SES increase [53,54]. Upward social mobility is paired with changes in workforce socio-demographics, as higher household income and educational attainment are linked to working in majority White geographical areas [26]. A lack of racial diversity can lead to increased racial discrimination, a lack of social support, and increased social isolation [27,28]—factors critical in elevating AL [8].

This study is not without limitations. First, because NHANES is a cross-sectional survey, neither causality nor temporality can be established. NHANES data also lacks reliable measures of experienced discrimination. We were also limited by the small sample of Black men with advanced college degrees, which precluded creating additional strata for more-specific analyses. Despite these limitations, the study has several strengths. First, NHANES sampling strategies permit weighting, allowing for more-stable estimations. The results have high validity due to NHANES’ use of a stratified multistage sampling scheme. Next, the inclusion of 20 years of NHANES survey data results in a dataset large and diverse enough to create an adequately sized subpopulation for analysis. Accordingly, both unweighted and weighted models revealed statistically significant results. Further, the inclusion of covariates allowed our analyses to account for confounding factors such as health and income that may affect AL and subsequent health outcomes. Lastly, we were not able to adjust for behavioral variables such as physical activity and alcohol consumption.

## 5. Conclusions

In conclusion, education is a key social determinant of health that has long been shown to beneficially influence adult health [55]. While our findings corroborate the broader literature on the association between AL and education level [24], we document this relationship for the first time in a population consisting exclusively of Black men. Although our findings suggest that among Black men higher educational attainment has a protective effect on biological processes, this effect was seen only among college graduates. While education alone did not explain the differences in AL by educational level, there is a critical link between educational attainment and health status. Future researchers must examine how the stress from Blacks experiencing discrimination and systemic racism in education may prevent Black men, specifically, from achieving optimal health, as well as examine how other measures of psychosocial stress including mental health, job security, and access to healthcare may play a role in AL among Black men. Our findings can inform effective interventions and policies to reduce health disparities among Black men by targeting SDoH that limit educational opportunities for Black men, while also calling attention to the chronic stress crisis that continues to confront and kill Black men in America.

## Figures and Tables

**Table 1:** Demographic characteristics, personal health, and medical conditions by educational status, among 4113 Black men, an estimated 8,116,464 US residents from the National Health and Examination Survey (NHANES) 1999–2018.

Characteristic	All	Less Than High School	High School/GED	Some College or Associates Degree	College Graduate	*p* Value ^2^
Participants (*n*)	4113	1198	1215	1116	584	
Estimated *n* ^1^ (Weighted %)	8,116,464	2,061,141 (25.4)	2,393,446 (29.5)	2,441,640 (30.1)	1,220,237 (15.0)	
Allostatic Load Total Score ^3,4^	2.04 (0.01)	2.15 (0.05)	2.01 (0.05)	1.98 (0.05)	2.05 (0.05)	0.14
% High Allostatic Load ^5,6^	18.9 (0.6)	19.0 (1.2)	19.2 (1.2)	18.6 (1.1)	18.4 (1.7)	0.9
Mean age in years ^4^	42.4 (0.3)	44.6 (0.6)	40.8 (0.5)	40.8 (0.6)	44.8 (0.5)	<0.01
Age group, years ^6^						
18–29	27.1 (0.9)	27.8 (1.4)	31.5 (1.5)	29.2 (1.7)	13.3 (1.8)	<0.01
30–39	19.0 (0.7)	13.8 (1.2)	18.9 (1.1)	21.2 (1.4)	23.7 (2.0)	
40–49	20.4 (0.7)	16.5 (1.3)	19.3 (1.2)	20.0 (1.3)	29.9 (2.2)	
50–59	17.0 (0.8)	18.4 (1.3)	15.5 (1.1)	17.0 (1.2)	17.6 (1.8)	
60–69	10.2 (0.4)	13.0 (0.9)	9.3 (0.6)	8.6 (0.8)	10.6 (0.9)	
70+	6.2 (0.3)	10.5 (0.8)	5.4 (0.5)	4.1 (0.5)	4.9 (0.7)	
Family Poverty to Income Ratio ^4^	2.45 (0.04)	1.69 (0.05)	2.21 (0.06)	2.74 (0.06)	3.62 (0.03)	<0.01
Current Smoker Status ^6^	30.0 (0.9)	39.8 (1.7)	33.2 (1.4)	27.2 (1.4)	12.6 (1.4)	<0.01
Any Cancer History ^6,7^	4.1 (0.3)	4.6 (0.5)	3.2 (0.5)	4.2 (0.6)	4.9 (0.7)	0.16
Angina ^6^	1.2 (0.2)	0.9 (0.3)	1.3 (0.3)	1.2 (0.3)	1.7 (0.5)	0.60
Ever Congestive Heart Failure ^6^	2.5 (0.2)	3.1 (0.5)	2.2 (0.5)	2.4 (0.4)	1.7 (0.5)	0.28
Ever Heart Attack ^6^	3.4 (0.3)	5.5 (0.7)	2.8 (0.5)	2.7 (0.5)	2.7 (0.7)	<0.01

^1^ Estimated using sampling weights from National Health and Nutrition Examination Survey (NHANES). ^2^ *p* values determined using Rao-Scott Chi-Square tests for categorical variables and weighted Wald F-tests for continuous variables. ^3^ Allostatic load total score was calculated as sum total of components based on high-risk thresholds: albumin, BMI, creatinine clearance, diastolic blood pressure, glycohemoglobin, systolic blood pressure, total cholesterol, triglycerides. Score ranges from 0 to 8. ^4^ Presented as weighted mean (standard error) for continuous variables. ^5^ High allostatic load defined as total allostatic load score greater than or equal to 4. ^6^ Presented as weighted column proportion (standard error). ^7^ Defined as self-reported response to ever being diagnosed by a doctor or health professional of any cancer or malignancy.

**Table 2:** Prevalence ratios (PRs) ^1^ and associated 95% confidence intervals (CIs) to examine the association between education and high allostatic load prevalence with National Health and Nutrition Examination Surveys (NHANES), 1999–2018 (*n* = 4113) ^2^.

Education Level	^3^ No. (Weighted %)	Crude	^4^ Model 1	^5^ Model 2	^6^ Model 3
Less than High School (Referent)	231(25.6)	1.00 (Referent)	1.00 (Referent)	1.00 (Referent)	1.00 (Referent)
High School/GED	236 (30.1)	1.01 (1.00–1.03)	1.09 (1.07–1.11)	1.10 (1.08–1.12)	1.11 (1.09–1.14)
Some College or Associates Degree	230 (29.6)	0.98 (0.97–0.99)	1.03 (1.01–1.04)	1.06 (1.04–1.08)	1.06 (1.04–1.08)
College Graduate	120 (14.7)	0.97 (0.93–1.01)	0.87 (0.84–0.90)	0.92 (0.89–0.96)	0.90 (0.87–0.93)

^1^ Prevalence Ratios for high allostatic load are estimated using modified Poisson regression with robust variance estimation and accounting for NHANES weighting. Confidence intervals estimated using delete-1 jackknife method accounting for complex statistical weighting, cluster, and strata. ^2^ Study population: 4113 Black Men, using NHANES weighting an estimated 8,116,464 participants across the US. ^3^ Number of participants with high allostatic load per educational level (weighted % represents the weighted proportion within each educational level given high allostatic load). There are 817 total Black men with high allostatic load. ^4^ Model 1: Adjusted for age only. ^5^ Model 2: Additionally, adjusted for poverty to income ratio. ^6^ Model 3: Additionally, adjusted for smoker status, ever congestive heart failure, ever heart attack, cancer, and angina.

**Table 3:** Prevalence Association between education and adjusted mean estimates of allostatic load and Associated 95% confidence intervals (CIs), Among 4113 participants, using NHANES weighting an estimated 8,116,464 US Black men.

		Adjusted Mean Estimates, (95% CI)		
Education Level	Crude	Model 1	Model 2	Model 3
Less than High School	2.16 (2.08–2.25)	2.19 (2.11–2.27)	2.16 (2.08–2.25)	2.19 (2.10–2.29)
High School/GED	2.02 (1.94–2.10)	2.11 (2.03–2.19)	2.02 (1.94–2.10)	2.03 (1.94–2.12)
Some College or Associates Degree	2.08 (2.00–2.17)	2.11 (2.03–2.20)	2.08 (2.00–2.17)	1.97 (1.89–2.07)
College Graduate	2.16 (2.04–2.28)	2.05 (1.94–2.16)	2.16 (2.04–2.28)	2.06 (1.94–2.18)

Mean estimates of allostatic load are modeled using Negative Binomial Regression and accounting for NHANES weighting. Model 1: Adjusted for age only. Model 2: Additionally adjusted for poverty to income ratio. Model 3: Additionally adjusted for smoker status, ever congestive heart failure, and ever heart attack.

## Data Availability

Centers for Disease Control and Prevention (CDC). National Center for Health Statistics (NCHS). National Health and Nutrition Examination Survey Data. Hyattsville, MD: U.S. Department of Health and Human Services, Centers for Disease Control and Prevention, (1999–2018) (https://www.cdc.gov/nchs/nhanes/index.htm (accessed on 2 March 2022)).

## Appendix A

**Table A1:** Weighted distribution of allostatic load components comparing subsample of non-Hispanic Black men vs. entire NHANES sample.

Allostatic Load Component	Mean (SE)	Min	Median (Q1, Q3) ^1^	Max
Serum Albumin (g/dL)				
non-Hispanic Black men (*n* = 4113)	4.28 (0.007)	1.90	4.24 (4.03, 4.46)	5.40
Entire NHANES Sample (*n* = 41,169)	4.30 (0.004)	1.90	4.25 (4.03, 4.47)	5.70
Body Mass Index (kg/m^2^)				
non-Hispanic Black men (*n* = 4113)	28.6 (0.117)	14.2	27.5 (23.8, 32.1)	74.1
Entire NHANES Sample (*n* = 41,169)	28.6 (0.066)	13.2	27.5 (23.9, 32.0)	130.2
Creatinine (μmol/L)				
non-Hispanic Black men (*n* = 4113)	99 (0.95)	46	92 (81, 105)	1574
Entire NHANES Sample (*n* = 41,169)	78 (0.21)	18	74 (62, 88)	1574
Diastolic Blood Pressure (mmHg)				
non-Hispanic Black men (*n* = 4113)	74 (0.30)	4	73 (64, 81)	128
Entire NHANES Sample (*n* = 41,169)	71 (0.14)	4	70 (63, 78)	134
Glycohemoglobin ^2^ (%)				
non-Hispanic Black men (*n* = 4113)	5.77 (0.020)	3.30	5.50 (5.20, 5.86)	16.5
Entire NHANES Sample (*n* = 41,169)	5.55 (0.008)	2.00	5.33 (5.08, 5.63)	18.0
Systolic Blood Pressure (mmHg)				
non-Hispanic Black men (*n* = 4117)	127 (0.33)	78	124 (114, 135)	236
Entire NHANES Sample (*n* = 41,169)	123 (0.17)	66	119 (110, 130)	270
Total cholesterol (mg/dL)				
non-Hispanic Black men (*n* = 4113)	187 (0.81)	80	183 (158, 212)	525
Entire NHANES Sample (*n* = 41,169)	195 (0.39)	59	191 (165, 220)	727
Serum triglycerides (mg/dL)				
non-Hispanic Black men (*n* = 4113)	122 (1.81)	20	93 (64, 145)	1605
Entire NHANES Sample (*n* = 41,169)	149 (1.12)	9	116 (78, 177)	6057

^1^ Q1 represents the first quartile, Q3 represents the third quartile. ^2^ Glycohemoglobin % (Hemoglobin A1c), is a diabetes test that reflects plasma glucose for the previous 120 days.
